# *KP177R*-based visual assay integrating RPA and CRISPR/*Cas12a* for the detection of African swine fever virus

**DOI:** 10.3389/fimmu.2024.1358960

**Published:** 2024-04-09

**Authors:** Haorui Luan, Shujuan Wang, Lin Ju, Tong Liu, Haoyue Shi, Shengqiang Ge, Shijin Jiang, Jiaqiang Wu, Jun Peng

**Affiliations:** ^1^College of Veterinary Medicine, Shandong Agricultural University, Taian, China; ^2^East China Scientific Experimental Station of Animal Pathogen Biology of Ministry of Agriculture and Rural Affairs, Shandong Provincial Key Laboratory of Animal Biotechnology and Disease Control and Prevention, Taian, China; ^3^China Animal Health and Epidemiology Center, Qingdao, China; ^4^Institute of Animal Science and Veterinary Medicine, Shandong Academy of Agricultural Sciences, Jinan, China

**Keywords:** African swine fever virus, KP177R gene, recombinase polymerase amplification, fluorescein-labeled, biotin-labeled, CRISPR/Cas12

## Abstract

**Introduction:**

Early detection of the virus in the environment or in infected pigs is a critical step to stop African swine fever virus (ASFV) transmission. The p22 protein encoded by ASFV *KP177R* gene has been shown to have no effect on viral replication and virulence and can serve as a molecular marker for distinguishing field virus strains from future candidate *KP177R* deletion vaccine strains.

**Methods:**

This study established an ASFV detection assay specific for the highly conserved ASFV *KP177R* gene based on recombinase polymerase amplification (RPA) and the CRISPR/Cas12 reaction system. The *KP177R* gene served as the initial template for the RPA reaction to generate amplicons, which were recognized by guide RNA to activate the trans-cleavage activity of *Cas12a* protein, thereby leading to non-specific cleavage of single-stranded DNA as well as corresponding color reaction. The viral detection in this assay could be determined by visualizing the results of fluorescence or lateral flow dipstick (LFD) biotin blotting for color development, and was respectively referred to as fluorescein-labeled RPA-CRISPR/*Cas12a* and biotin-labeled LFD RPA-CRISPR/*Cas12a*. The clinical samples were simultaneously subjected to the aforementioned assay, while real-time quantitative PCR (RT-qPCR) was employed as a control for determining the diagnostic concordance rate between both assays.

**Results:**

The results showed that fluorescein- and biotin-labeled LFD *KP177R* RPA-CRISPR/*Cas12a* assays specifically detected ASFV, did not cross-react with other swine pathogens including PCV2, PEDV, PDCoV, and PRV. The detection assay established in this study had a limit of detection (LOD) of 6.8 copies/μL, and both assays were completed in 30 min. The *KP177R* RPA-CRISPR/*Cas12a* assay demonstrated a diagnostic coincidence rate of 100% and a kappa value of 1.000 (p < 0.001), with six out of ten clinical samples testing positive for ASFV using both *KP177R* RPA-CRISPR/*Cas12a* and RT-qPCR, while four samples tested negative in both assays.

**Discussion:**

The rapid, sensitive and visual detection assay for ASFV developed in this study is suitable for field application in swine farms, particularly for future differentiation of field virus strains from candidate *KP177R* gene-deleted ASFV vaccines, which may be a valuable screening tool for ASF eradication.

## 1 Introduction

African swine fever (ASF), an acute, febrile, and virulent infectious disease in pigs caused by the ASF virus (ASFV), is clinically characterized by high fever, hemorrhage, and can infect different breeds of pigs and wild boars of diverse ages, with a lethality rate of up to 100% (1). The virus can be transmitted through soft tick of Ornithodoros species (1) or through direct contact with infected pigs (2). Owing to its serious harm, it should be reported to the World Organization for Animal Health (WOAH) and is a class I animal infectious disease in China; thus, its prevention and control is relevant worldwide. Since its introduction in China in 2018, the wide spread of ASF has dealt a fatal blow to the Chinese pig industry (3). ASFV belongs to the genus *Asfivirus* in the family *Asfarviridae*, has a double-stranded DNA molecule of 170–190 kb (1, 4), contains 150–167 open reading frames (5), and encodes nearly 200 proteins (1, 6). ASFV is an enveloped virus, and the viral particle comprises five parts: the outer capsid, capsid, inner capsid, nucleocapsid, and inner core (7). ASFV is structurally complex, and researchers do not yet fully understand the functions of its many proteins. To date, no credible commercial vaccine has been developed worldwide, and there are no effective treatments for infected pigs. Currently, adopting strict biosecurity prevention measures to block viral infection or eliminate outbreaks through rapid and accurate virus detection and the full culling of sick pigs is a proven method. In this regard, early detection of the virus in the environment, equipment, and infected pigs is critical for its elimination.

ASF surveillance can be performed by pathogen screening and antibody detection. In terms of antibody testing, in pigs infected with ASFV, immunoglobulin M (IgM) and IgG can be detected 4 and 6–8 days post-infection, respectively (8). Antibodies can circulate concurrently with the virus for up to six months after infection (8, 9) and remain detectable for years after the first exposure. IgM is produced early, but in small amounts, whereas large amounts of immunoglobulins, such as IgG, take approximately one week to be produced. These delays are fatal and unacceptable for the early detection of the virus because by the time antibodies can be detected, the virus has been infecting, replicating, and proliferating for at least one week and has been discharged into the environment. Therefore, for early detection of the virus, the environment or suspected infected pigs should be tested for pathogens in a timely manner, for example, by sampling and testing every day or every half day.

Commonly used methods for ASFV detection include the polymerase chain reaction (PCR), real-time quantitative PCR (RT-qPCR), hemadsorption test (HAT) and viral antigen (10). Among them, PCR demonstrated a LOD of 40 or 60 DNA copies per microliter, respectively (11, 12); and RT-qPCR was 18 DNA copies (13). The sensitivity of HAT was significantly lower than that of the other two methods; however, HAT was the reference standard for the diagnosis of ASF by WOAH (14). The abovementioned detection methods rely on a variety of laboratory instruments and equipment and have complicated operation steps that require trained professional technicians, which dramatically limits their application in the field of pig farms, making it challenging to meet the requirements of rapid virus detection. Therefore, it is crucial to develop a simple ASFV detection method that requires few reagents, does not require specialized instrumentation, has high sensitivity, and is suitable for on-site use in farms to immediately screen ASFV pathogens. The clustered regularly interspaced short palindromic repeats (CRISPR)/Cas system is widely used for gene editing. *Cas12a* has three proteins with efficient specific cleavage activity induced by cis-cleavage supplemented by guide RNA (gRNA), which can be used for pathogenic gene detection in various diseases. Recombinase polymerase amplification (RPA) is a temperature-controlled rapid amplification technique using recombinase and polymerase as the core components and single-stranded binding proteins as the auxiliary components, which can rapidly amplify the target fragment in a short period (e.g., 30 min) at a constant temperature (e.g., 37°C). The combined application of CRISPR/Cas and RPA can establish a rapid ASFV detection assay that meets the above requirements.

ASFV encodes a variety of proteins, and the functions of some proteins have been confirmed, but most of which are still unclear. For example, the p54 and p72 structural proteins are integral to viral particles (15, 16), whereas the viral *pE402R* (CD2v) homolog is the only marker molecule of the outside-virus structure (17). Non-structural proteins encoded by multigene families, such as *A137R, E120R, MGF360-9L, and MGF505-7R*, function as inhibitors of type I interferons (18–21), and *MGF505-7R and MGF360-9L*, which are the major virulence factors of the virus (20, 21). The p22 protein applied in this study, encoded by *KP177R*, is a transmembrane structural protein transcribed early in ASFV (22, 23). This protein is a component of the inner membrane, is transiently expressed on the surface of infected cells, and is closely associated with outer envelope formation (17). Recently, it has been reported that the p22 protein is a target of interactions with several host proteins that exert viral endocytosis through the cyclic GMP-dependent protein kinase (cGMP-PKG), cAMP, and AMP-activated protein kinase (AMPK) signaling pathways (24). The recent study has demonstrated that p22 protein knockdown exerts no discernible impact on virus replication *in vitro* or in cells, does not influence virulence, and is dispensable for the viral fundamental functions (25).. Therefore, based on the role played by the p22 protein in viral replication and virulence, the *KP177R* gene is promising to be used in the preparation of ASFV gene deletion vaccines (25). It can serve as a molecular marker for distinguishing field virus strains from future candidate gene deletion vaccines strains, just as the PRV gene deletion vaccines did.

Targeting the *KP177R* gene, a visual ASFV detection assay based on CRISPR/*Cas12a* combined with RPA is a valuable tool for screening viruses in the environment or infected pigs as early as possible. More important, it may be a good tool to distinguish future candidate *KP177R* gene-deleted ASFV vaccines from field virus strains.

## 2 Materials and methods

### 2.1 Viral strains and nucleic acids

The ASFV *KP177R* gene sequence used in this study was derived from ASFV strain China/2018/AnhuiXCGQ (GenBank accession no. MK128995.1), and the nucleotide fragment of the gene was synthesized by Tsingke Genomics (Tsingke, Beijing, China). The porcine reproductive and respiratory syndrome virus (PRRSV) strain CSR1801 (GenBank accession No. OM743305.1) and porcine circovirus type 2 (PCV2) strain CSR2301 (GenBank accession No. OQ865055.1) were isolated and stored in the laboratory. cDNA from the porcine epidemic diarrhea virus (PEDV) CHM2013 (GenBank accession No. KM887144.1), porcine deltacoronavirus (PDCoV) CHN-HN-1601 (GenBank accession No. MG832584.1), and the DNA from pseudorabies virus (PRV) HB1201 strains (GenBank accession No. KU057086.1) were kindly provided by the College of Veterinary Medicine of China Agricultural University, Beijing, China.

### 2.2 Construction of *Cas12a* expression plasmid

The 6His-MBP-TEV-huLb*Cas12a* plasmid (Addgene, Watertown, MA, USA) was used as a template for designing *Cas12a* amplification primers with homology arms (Primer Premier v. 5.0; PREMIER Biosoft, Palo Alto, CA, USA): *Cas12a*-F/*Cas12a*-R (**Table 1**), carried *Bam*HI and *Xho*I cleavage sites at the front and back ends, respectively. PCR amplification of *Cas12a* gene, in a 25 μL of reaction system, contained 2 μL of *Cas12a* plasmid template, 1 μL of *Cas12a*-F, 1 μL of *Cas12a*-R, 12.5 μL of 2 × Es Taq Master Mix, and 8.5 μL of ddH_2_O; the reaction program was: 95°C pre-denaturation for 5 min, 95°C denaturation 30 s, and 56°C annealing for 30 s, 72°C extension for 120 s, 30 cycles of reaction, and 4°C. At the end of the PCR reaction, agarose gel electrophoresis was performed, the target genes were recovered and sequenced for identification, and the recovered target genes were stored at –20°C. The pET-28a vector (Tiangen Biotech, Beijing, China) was subjected to *Bam*HI and *Xho*I double enzyme digestion, agarose gel electrophoresis recovered pET-28a target fragment, the vector fragment and *Cas12a* fragment with added homologous recombination site were subjected to ligation reaction under the action of T4 DNA ligase, and ligated products were transformed to the DH5α competent cells, and the monoclonal clones with kanamycin resistance were selected. The *Cas12a* prokaryotic expression plasmid with the correct gene sequence was obtained, named pET-28a-*Cas12a*, and stored at –80°C.

**Table 1:** Primers used in this study.

Primers	Primer sequences (5′→3′)	Production length
*KP177R*-F	ATGTTTAATATTAAAATGACAATTTCTACATTGC	534bp
*KP177R*-R	TTATGCATGTTTATGATTTCTAGGTAAGGC	
*Cas12a*-F	CAGCAAATGGGTCGCGGATCCATGAGCAAGCTGGAGAAGTTTACA	3684bp
*Cas12a*-R	GTGGTGGTGGTGGTGCTCGAGGTGCTTCACGCTGGTCTGGG	
RPA-F1	GACAATTTCTACATTGCTTATTGCTCTTAT	120bp
RPA-R1	CCACAATCTTTATCTACTTTACAGACCTTT	
RPA-F2	ATGACAATTTCTACATTGCTTATTGCTCTT	428bp
RPA-R2	GTTCCCTCCTTTTCATCACCAACATATTCC	
RPA-F3	ATGACAATTTCTACATTGCTTATTGCTCTT	424bp
RPA-R3	CCTCCTTTTCATCACCAACATATTCCCAAC	
RPA-F4	AAATGACAATTTCTACATTGCTTATTGCTC	476bp
RPA-R4	ACCATATTTAAGAACCGGGTGATGTGGATT	

### 2.3 Expression and purification of *Cas12a* protein

The recombinant plasmid pET-28a-*Cas12a* was transformed into BL21 (DE3) competent cells (ThermoFisher Scientific, Shanghai, China), and the monoclonal colonies were picked and inoculated with kanamycin-resistant LB liquid medium. IPTG was added to induce protein expression when the OD_600_ value reached 0.6~0.8. The IPTG was set at 100, 300, 500, and 700 μM, the temperature was set at 18 and 37°C, and the incubation was performed at 5.37 g for 4, 6 and 8 h, so as to screen the optimal conditions for induced expression.

Under the optimal expression conditions described above, pET-28a-*Cas12a* was induced in large quantities in the liquid LB medium. The bacterial fluid was collected, centrifuged at 7,104 g for 10 min, the bacterial precipitate was collected, and the bacterial lysis solution and protease inhibitor (Beyotime, Shanghai, China) were added successively, and then the bacterial bodies were broken by ultrasonication (300 W, 3 s bursts with 6 s pauses, for 30 min on ice), the precipitate was collected by centrifugation and resuspended with 8 M urea solution, and the supernatant was centrifuged at 13,400 g for 20 min and filtered through a 0.45 μm filter to remove impurities. The *Cas12a* protein was purified according to Ni-agarose instructions (CWBio, Beijing, China). Briefly, the inclusion body protein solution was loaded into the sampling column, and an elution buffer was added to elute the target protein. The purified *Cas12a* protein was collected, and the protein concentration was determined by Micro BCA Protein Assay Kit (CWBio), stored at –80°C.

### 2.4 SDS-PAGE and western blot

Purified *Cas12a* protein was separated by SDS-PAGE and identified by Western blotting. The main steps were as follows: the *Cas12a* protein was added to a 12% SDS-PAGE separation gel and electrophoresed for 20 min at 80 V, and the lower layer of the gel was electrophoresed for 90 min at 120 V. The protein separation gel was transferred at a current of 200 mA for 1.5 h, and the protein on the gel was transferred to a PVDF membrane. Then it was washed with phosphate buffered saline with tween-20 (PBST), blocked with 5% dry milk in PBST, and washed again before adding anti-His tag mouse monoclonal antibody (Beyotime) as primary antibody (1:3,000) and incubated at 4°C overnight. After washing, HRP-labeled goat anti-mouse IgG (Jackson ImmunoResearch, West Grove, PA, USA) was added as a secondary antibody (1:5,000) and incubated at room temperature for 2 h. After washing, an enhanced chemiluminescence detection kit (ThermoFisher Scientific) was used to blot the target protein and images were captured using a ChemiDocTM MP Imaging System (Bio-Rad Laboratories, Hercules, CA, USA).

### 2.5 gRNA and ssDNA design and synthesis

The *KP177R* gene of the ASFV Anhui XCGQ strain was used as a target template to design a guide RNA (gRNA) for the *Cas12a* protein with the sequence UAAUUUCUACUAAGUGUAGAUUUACAUUUGCUUAUUUGCUCUUUA, which was localized at the 27th to 46th bases of the *KP177R* gene. The sequence specificity of this gRNA was analyzed using the NCBI online BLAST tool (https://www.ncbi.nlm.nih.gov/tools/primer-blast/) to determine whether the gRNA does not bound a non-target *KP177R* fragment or a nucleotide paralog in the GenBank database. The gRNA was synthesized and diluted to a concentration of 10 μM and stored at –80°C. The *Cas12a* protein first needs to bind to a specific gRNA, and the cleavage properties of the *Cas12a* protein can be activated if the *Cas12a* protein recognizes the target DNA sequence and cleaves the single-stranded DNA. To detect the target DNA sequence, for *Cas12a* protein and *KP177R* target sequence, TTATT was used as the basic ssDNA sequence. A FAM fluorescent chromogenic element was added to the 5’ end of the ssDNA reporter, and a black hole quencher 1 (BHQ1) element was added to the 3’ end. The ssDNA reporter had a sequence of 5’-FAM-TTATT-BHQ1-3’ and this fluorescent reporter was named ssDNA-F. A FAM fluorescent chromogenic element was added to the 5’ end of the ssDNA reporter and a biotin was added to the 3’ end. The ssDNA reporter was sequenced as 5’-FAM-TTATT-Biotin-3’, and the biotin reporter was named ssDNA-B. These two reporters were synthesized using Tsingke genomics (Tsingke).

### 2.6 RPA primer design and *KP177R* RPA assay

The complete *KP177R* gene fragment of ASFV was synthesized based on the full gene sequence of the ASFV Anhui XCGQ strain, and PCR primers *KP177R*-F/*KP177R* (**Table 1**) were used to amplify the gene fragments. The PCR reaction conditions were 95°C pre-denaturation for 5 min, 95°C denaturation for 30 s, 55°C annealing for 30 s, 72°C extension for 45 s, and 30 cycle reactions. The PCR products were transformed into *Escherichia coli* competent cells DH5α, single colonies were picked for verification, and the recombinant plasmid was named pMD18-T-*KP177R* and stored for subsequent experiments. RPA amplification primers should be designed according to the binding site of the gRNA and must contain the target sequence to be detected. In this study, four pairs of RPA amplification primers (RPA-F1/RPA-R1, RPA-F2/RPA-R2, RPA-F3/RPA-R3, and RPA-F4/RPA-R4) were designed and synthesized (Tsingke); their sequences are shown in **Table 1**, which amplify 120, 428, 424 and 476 bp length fragments of the *KP177R* gene, respectively. Using the above RPA-F/RPA-R primers, gene amplification was performed using the pMD18-T-*KP177R* plasmid as a template according to the instructions of the DNA Isothermal Rapid Amplification Kit (Amp-Future, Weifang, China). The main steps were as follows: 29.4 μL Buffer A, 2 μL RPA-F (10 μM), 2 μL RPA-R (10 μM), 12.1 μL ddH_2_O, 2 μL pMD18-T-*KP177R* (420 ng/μL), and 2.5 μL Buffer B were added to the system, which was mixed thoroughly, and reacted for 30 min at 37°C in a metal bath. At the end of the reaction, Tris-saturated phenol/chloroform/isoamyl alcohol (25:24:1) extraction solution was added, mixed thoroughly at a 1:1 ratio, and centrifuged at 13,400 g for 5 min. The supernatant of the uppermost of the three layers was collected and the RPA amplification product, was analyzed by agarose gel electrophoresis.

### 2.7 *KP177R* RPA-CRISPR/*Cas12a* assay

The *KP177R* RPA-CRISPR/*Cas12a* assay system consisted of the RPA amplification product of the target dsDNA *KP177R* gene, *Cas12a* protein, NEBuffer 2.1, ssDNA reporter, gRNA, and ddH_2_O. Fluorescence intensity was measured using a 96-well microplate reader to determine whether the complete reaction system was stable. The assay system was set to 50 μL, containing 5 μL of NEBuffer 2.1, 5 μL of *KP177R* RPA amplification product, 5 μL of *Cas12a* protein (2.5 μg/μL), 2.5 μL of gRNA (10 μM), 5 μL of fluorescein-labeled ssDNA reporter (ssDNA-F, 10 μM), and 27.5 μL of ddH_2_O. At the same time, four groups of independent reaction systems lacking either the *KP177R* RPA amplification product, *Cas12a* protein, gRNA or ssDNA reporter were used as controls. The prepared assay system was placed in a microplate reader, the excitation and emission wavelength were set at 485 and 521 nm, respectively, and the reaction was performed at 37°C for 1 h. Fluorescence intensity was measured every 2 min and consecutive fluorescence values were collected. The above detection reactions were independently tested thrice and the results were analyzed statistically. In order to establish a visual fluorescence detection assay that is convenient for clinical field, the detection system of the above assay was set to 20 μL, containing 2 μL NEBuffer 2.1, 3 μL *KP177R* RPA amplification product, 1 μL *Cas12a* protein (2.5 μg/μL), 2 μL gRNA (10 μM), 1 μL ssDNA-F reporter (10 μM), and 11 μL ddH_2_O. The reaction system was mixed thoroughly and added into PCR tube strip (Roche, Shanghai, China). The reaction was performed in a metal bath at 37°C for 30 min, and the fluorescence status was observed and recorded with the naked eye under blue light at the end of the reaction.

Similar to the fluorescent reaction system described above, the fluorescent reporter ssDNA-F in the fluorescent reaction system was replaced with biotin-labeled reporter ssDNA (ssDNA-B), and the reaction product was blotted with the substrate on an LFD (Tiosbio, Beijing, China) to determine the target gene. With reference to the reagent ratios of the above fluorescent reaction system, a biotin reporter reaction system was prepared, containing 5 μL NEBuffer 2.1, 5 μL *KP177R* RPA amplification product, 5 μL *Cas12a* (2.5 μg/μL), 2.5 μL gRNA (10 μM), 5 μL ssDNA-B (10 μM), and 27.5 μL ddH_2_O. The reaction system was performed in a metal bath at 37°C, for 0, 10, 20, 30, 40, 50 min, and 60 min. Fifty microliters of the reaction product were added dropwise to LFD and incubated for 10 min. The blot densities of the detection T- and control C-lines were determined using Image J software (National Institutes of Health (NIH), Bethesda, MD, USA), and the corresponding T/C ratios were calculated for statistical analysis.

### 2.8 Optimization of the *KP177R* RPA-CRISPR/*Cas12a* assay reaction conditions

To determine the optimal reaction temperature and time for the *KP177R* RPA-CRISPR/*Cas12a* assay, the ssDNA-F reporter was selected to formulate the 50 μL assay system in a 96-well microplate. The reaction system was placed in a microplate reader, and different reaction temperatures were set: 20, 25, 30, 35, 37, 40, and 42°C and the fluorescence intensity was measured every 2 min for a total of 1 h, and the consecutive fluorescence intensities were recorded, and then the optimal reaction temperatures and reaction times of the fluorescein-labeled reaction system were determined after statistical analysis. Meanwhile, 20 μL of the reaction system was configured in a PCR tube strip, and the reaction was performed at the temperatures of 20, 25, 30, 35, 37, 40, and 42°C for 30 min, respectively, and the optimal reaction temperature was determined according to the fluorescence intensity under blue light irradiation.

To determine the optimal concentration of *Cas12a* protein and gRNA, the *Cas12a* protein concentration was set to 400, 200, 100, 50, and 25 nM, the gRNA concentration was set to 200, 100, 50, and 25 nM, and a checkerboard test was performed. The fluorescent reporter ssDNA-F was selected to formulate the assay system, which was performed for 1 h at 37°C, and the fluorescence intensity was measured at intervals of 2 min, and a continuous report of the fluorescence value was obtained. At the end of the reaction, the Heatmap command in GraphPad software (Boston, MA, USA) was used to create graphs and for comparative analysis to determine the optimal *Cas12a* protein and gRNA reaction concentrations.

To determine the optimal reaction temperature of the biotin-labeled reaction system, according to the optimal reaction concentration of *Cas12a* protein and gRNA determined by the above test, biotin-labeled reporters were selected to formulate the CRISPR/Cas assay: 5 μL NEBuffer 2.1, 5 μL *KP177R* RPA amplification product, 5 μL *Cas12a* (100 nM), 2.5 μL gRNA (100 nM), 5 μL ssDNA-B (10 μM), and 27.5 μL ddH_2_O, placed in a metal bath, and set different temperatures of 20, 25, 30, 35, 37, 40, and 42°C, and reacted for 20 min. Image J software was used to determine the color densities of the LFD detection T- and control C-lines and to calculate the corresponding T/C ratios. The biotin-labeled reaction system was configured according to the optimal reaction temperature determined from the above test to determine its optimal reaction time. The biotin-labeled reporter reaction system reacted at 40°C for 5, 10, 15, 20, and 25 min, and the color densities of the T- and C-lines were measured and the corresponding T/C ratios were calculated. To determine the optimal incubation time of the biotin-labeled reaction products with the test strips, the biotin-labeled reporter detection system was configured according to the optimal reaction temperature and reaction time determined from the above tests. The reaction products were added dropwise to the test strips after a 20 min reaction at 40°C, and the incubation times were set to 2, 4, 6, 8, and 10 min. The color densities of the T- and C-lines were determined and the corresponding T/C ratios were calculated.

### 2.9 Specificity evaluation of the *KP177R* RPA-CRISPR/*Cas12a* assay

To assess the specificity of the assay, nucleic acids of PRRSV, PCV2, PEDV, PDCoV, and PRV were used as controls. The nucleic acids of ASFV and the above five types of viruses were simultaneously amplified isothermally using the RPA established in this study, and then the amplified products were measured using the fluorescein- and biotin-labeled reporter reaction systems to determine the specificity of this *KP177R* RPA-CRISPR/*Cas12a* for the detection of the target gene.

### 2.10 Evaluation of the analytical sensitivity of the *KP177R* RPA-CRISPR/*Cas12a* assay

The concentration of the positive plasmid pMD18-T-*KP177R* of the sample to be detected, *KP177R*, was 6.83 × 10^10^ copies/μL, which was tenfold diluted to 6.83 × (10^8^, 10^7^, 10^6^, 10^5^、10^4^, 10^3^, 10^2^, 10^1^ and 10^0^) to obtain different concentrations of the sample DNA to be detected. Different concentrations of sample DNA were used as templates for RPA amplification, and the RPA amplification products were used as target dsDNA for CRISPR/*Cas12a* detection. The sensitivity of this CRISPR/*Cas12a* assay to fluorescein- and biotin-labeled reporter reaction systems was determined by observing the fluorescence color development and the T/C ratios of the LFD, respectively.

### 2.11 Evaluation of the reproducibility analysis of the *KP177R* RPA-CRISPR/*Cas12a* assay

The RPA amplification products of strongly, moderately, and weakly positive *KP177R* DNA, 6.83 × 10^7^, 6.83 × 10^5^, and 6.83 × 10^3^ copies/μL, were used as the target DNA to evaluate the CRISPR/*Cas12a* fluorescein- and biotin-labeled reaction systems reproducibility. Three independent runs, each with three technical replicates, were conducted under optimal conditions on the three DNA templates extracted monthly for three months.

### 2.12 Evaluation of the detection performance of the *KP177R* RPA-CRISPR/*Cas12a* assay

Ten *KP177R* gene fragments amplified from clinical samples of suspected ASF collected in 2020-2022 by the National Reference Laboratory for ASF, China Animal Health and Epidemiology Center (Qingdao, China) were used to assess the performance of the *KP177R* RPA-CRISPR/*Cas12a* assay. *KP177R* RPA-CRISPR/*Cas12a* and RT-qPCR were used to detect these gene fragments simultaneously. The RT-qPCR assay recommended by WOAH was performed as previously described (13). The *KP177R* RPA-CRISPR/*Cas12a* detection results were compared to those acquired from the parallel RT-qPCR assay to calculate the diagnostic coincidence rates corresponding to the two assays.

### 2.13 Statistical analyses

The data were expressed as the mean ± standard deviation (X ± SD). Duncan’s multi-sample test was used to analyze the differences between the groups, and SPSS 19.0 was used for statistical analysis. *p* < 0.05 was considered statistically significant.

## 3 Results

### 3.1 Expression, purification and characterization of *Cas12a* protein

**Figure 1:**
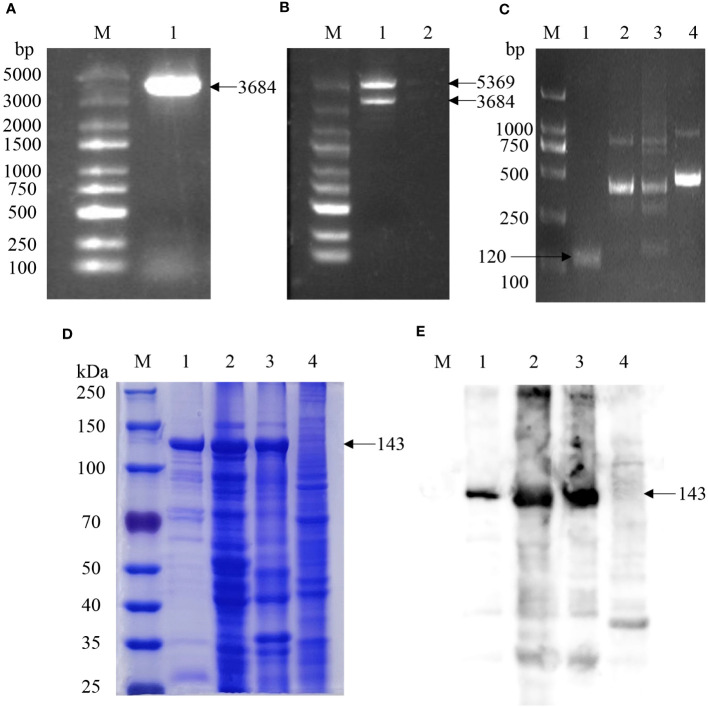
Expression and identification of the *Cas12a* protein and RPA amplification of *KP177R* gene fragment. **(A)** *Cas12a* gene amplification: M, 5000 bp marker; 1, PCR amplification of *Cas12a* gene. **(B)** Identification of pET-28a-*Cas12a* by double enzymes digestion: M, 5000 bp marker; 1, pET-28a-*Cas12a* was digested by *Bam*HI and *Xho*I enzymes; 2, negative control. **(C)** Amplification of *KP177R* gene using RPA: M, 2000 bp marker; 1−4, amplification products by RPA F1/R1, RPA F2/R2, RPA F3/R3 and RPA F4/R4 primers, respectively. **(D)** SDS-PAGE analysis of *Cas12a* protein: M, 250 kDa marker; 1, purified *Cas12a* protein after lysis of the target gene transforming bacteria; 2, unpurified supernatant protein after lysis of the target gene transforming bacteria; 3, unpurified precipitated protein after lysis of the target gene transforming bacteria; 4, unpurified protein after lysis of the empty vector transforming bacteria. **(E)** Western blot identification of *Cas12a* protein: Western blot reaction with anti-His monoclonal antibody as primary antibody; M, 250 kDa marker; 1, purified *Cas12a* protein after lysis of target gene transforming bacteria; 2, unpurified supernatant protein after lysis of target gene transforming bacteria; 3, precipitated protein after lysis of target gene transforming bacteria; 4, unpurified protein after lysis of empty vector transforming bacteria.

The *Cas12a* gene was amplified by PCR using the 6His-MBP-TEV-huLb*Cas12a* plasmid as a template (**Figure 1A**) to construct the *Cas12a* expression plasmid pET-28a-*Cas12a*, which showed a band conforming to the molecular size of the target gene with a molecular weight of approximately 3,684 bp after double enzyme cleavage (*Bam*H I and *Xho* I) reaction (**Figure 1B**). The recombinant plasmid pET-28a-*Cas12a* was transfected into BL21 (DE3) competent cells to induce *Cas12a* protein expression. The culture temperature, IPTG concentration, and induction time were determined at 37°C for 6 hours with a concentration of 100μM IPTG. Bacteria were collected by centrifugation, and the supernatant and precipitate were subjected to SDS-PAGE after ultrasonic crushing, which showed that the *Cas12a* protein existed in the supernatant and precipitate of the crushed bacteria in the form of soluble and inclusion body expression (**Figure 1D**). Ni-affinity chromatography was used to obtain the purified *Cas12a* protein (**Figure 1D**). Western blotting identified the protein with a distinct band at a molecular weight of approximately 143 kDa, which was consistent with the molecular weight of the *Cas12a* target protein (**Figure 1E**).

### 3.2 *KP177R* gene amplification by RPA

The complete fragment of the ASFV *KP177R* gene was synthesized as a template and amplified it to construct the recombinant plasmid pMD18-T-*KP177R*, which was consistent with the original sequence of the gene according to sequencing review. The *KP177R* gene was amplified using four primer pairs (RPA-F/R1-F/R4). The RPA-F1/R1 primer was screened according to the agarose gel electrophoresis results, which confirmed that the amplification products matched the expected size of 120 bp (**Figure 1C**).

### 3.3 Optimal reaction conditions of the *KP177R* RPA-CRISPR/*Cas12a* assay

#### 3.3.1 Fluorescein-labeled reaction system

**Figure 2:**
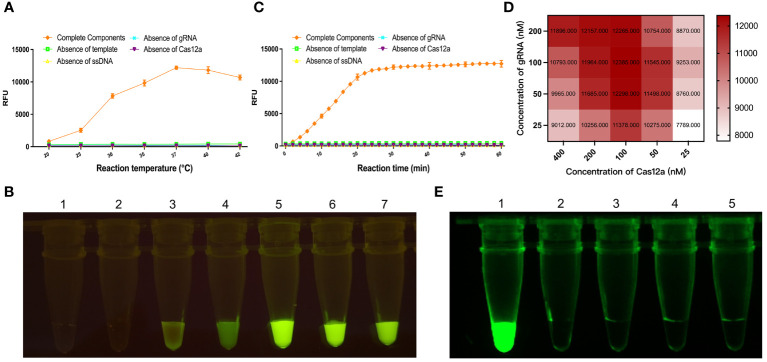
Optimization of parameters for the *KP177R* RPA-CRISPR/*Cas12a* fluorescein-labeled reaction system. Different reaction temperatures, reaction times, *Cas12a* concentrations, and gRNA concentrations were set, and experiments were performed to select the optimal reaction parameters. **(A)** Group containing complete components and control groups lacking gRNA, *KP177R*, *Cas12a*, and ssDNA, respectively, were formulated in 96-well microplates, and these systems were reacted at seven temperatures, 20, 25, 30, 35, 37, 40, and 42°C, respectively, and their corresponding fluorescence intensities were measured in a microplate reader. **(B)** Group containing complete components were configured in tubes strip to react at seven temperatures, 20, 25, 30, 35, 37, 40, and 42°C, respectively, and their fluorescence responses were recorded by blue light irradiation. **(C)** The above five groups were reacted for 10, 20, 30, 40, 50, and 60 min, respectively, and their corresponding fluorescence intensities were determined. **(D)** After determining the optimal reaction temperature and time, different *Cas12a* and gRNA concentration gradients were set, and reactions were performed to determine the corresponding *Cas12a* and gRNA concentrations at the highest value of RFU. **(E)** The reaction system to facilitate clinical ready-to-use was formulated in a 20 μL volume, reacted at 37°C for 30 min, and the performance of the reaction was determined by blue light irradiation at room temperature, with 1, 2, 3, 4, and 5 corresponding to the five groups mentioned above, respectively.

To determine *Cas12a* protein activity, the guiding effect of gRNA, and whether the gRNA-CRISPR/*Cas12a* detection system works, the gRNA-CRISPR/*Cas12a* fluorescence detection system was formulated in a 96-well microplate. After preparation, the system was placed in the microplates and reacted at different temperatures for 30 min for fluorescence intensity detection, and continuous fluorescence values were reported and statistically analyzed for each group. Among them, the fluorescence value of the complete component group containing the *KP177R* template gradually increased with time (**Figure 2A**). In contrast, the other four control groups lacking the *Cas12a* protein, ssDNA reporter, gRNA, and target dsDNA showed no fluorescence. The above results show that the gRNA-CRISPR/*Cas12a* fluorescence detection system has the highest fluorescence intensity at a reaction temperature of 37°C, which means that the *Cas12a* protein in the reaction system can efficiently cut the target gene at this temperature. Thus, it can be determined that the optimal reaction temperature for this detection system is 37°C. Meanwhile, the reaction was performed in tubes configured with the group containing the complete composition at different temperatures, respectively. The fluorescence intensity of this reaction system was highest at 37°C when irradiated with blue light (**Figure 2B**). The results of the above reactions in the microplates and tubes strip were consistent, and both showed that this gRNA-CRISPR/*Cas12a* fluorescein-labeled reaction system had the highest fluorescence intensity at 37°C. The above results show that the gRNA designed in this study can accurately identify the nucleic acid target sequences to be detected, the purified *Cas12a* protein has good nucleic acid cleavage activity for the target gene ASFV *KP177R*, and the ssDNA-F reporter has good fluorescence chromogenic activity. The gRNA-CRISPR/*Cas12a* assay system can work stably at 37°C and has the strongest fluorescence response, and the targeted cleavage of the ASFV *KP177R* gene shows specific green fluorescence.

To determine the optimal reaction time at 37°C reaction temperature, this study configured the gRNA-CRISPR/*Cas12a* reaction system in a 96-well microplate, and fluorescence intensity detection was performed at 2-min intervals, and the fluorescence intensity values of each group were analyzed. The results showed that the fluorescence intensity of the gRNA-CRISPR/*Cas12a* fluorescence detection system rose to its highest and maintained a plateau period after 30 min of reaction; therefore, the optimal reaction time for this detection system was chosen to be 30 min (**Figure 2C**). In order to determine the optimal reaction concentration of *Cas12a* protein and gRNA, *Cas12a* protein and gRNA were placed with different concentration gradients for checkerboard test, and the fluorescence intensity values were detected when the reaction was performed at 37°C for 30 min. As shown in **Figure 2D**, the highest fluorescence intensity was observed when the *Cas12a* protein concentration was 200 nM and gRNA concentrations were 100 nM; therefore, the optimal reaction concentrations of *Cas12a* protein and gRNA were 200 and 100 nM, respectively. The visual fluorescence reaction setup suitable for use in clinical *in situ* assays was a 20 μL system. The reaction was performed at 37°C for 30 min, and the results showed that the group containing the complete composition showed evident green fluorescence. In contrast, the other four control groups lacking *Cas12a* protein, ssDNA molecule, gRNA, and target dsDNA showed no fluorescence (**Figure 2E**).

#### 3.3.2 Biotin-labeled reaction system

**Figure 3:**
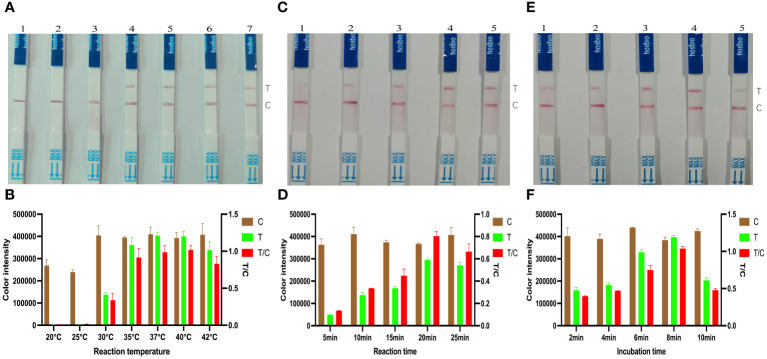
Optimization of *KP177R* RPA-CRISPR/*Cas12a* biotin-labeled reaction system. **(A)** Configuration of the biotin-labeled reaction system at 20, 25, 30, 35, 37, 40, and 42°C, marked with dipsticks 1−7, respectively, performed to find its optimal reaction temperature. **(B)** Image J densitometry of the signal intensities at the T- and C-lines in the LFD and calculation of their relative densitometric ratios for different reaction temperatures. **(C)** The biotin-labeled reaction system was configured and the reaction was performed to find its optimal reaction time at 20°C temperature for 5, 10, 15, 20, and 25 min, marked with dipsticks 1−5, respectively. **(D)** Image J densitometry of the signal intensities at the T- and C-lines in the LFD and calculation of their relative densitometric ratios for different reaction times. **(E)** The biotin-labeled reaction system was configured and reacted for 20 min at a temperature of 20°C, and the reaction solution was added dropwise to the LFD and incubated for 2, 4, 6, 8, and 10 min, marked with dipsticks 1−5, respectively, to find the optimal incubation time **(F)** Image J densitometry of the signal intensities at the T- and C-lines in the LFD and calculation of their relative densitometric ratios for different incubation times.

A biotin reporter reaction system was used to detect the target gene *KP177R*, which was reacted at different temperatures, respectively. An appropriate amount of reaction solution was added dropwise to the LFD after 20 min, and the color densities of the T and C line were determined by using the Image J software to calculate the corresponding T/C ratios. The results showed that the optimal reaction temperature for this biotin reporter reaction system was 40°C (**Figures 3A, B**). Similarly, with the above determination of 40°C as the optimal reaction temperature, 50 μL of the reaction solution was added dropwise to the LFD at various durations and the corresponding T/C ratios were calculated. The results showed that the highest T/C ratios were obtained at 20 min (**Figures 3C, D**). After the above reaction system was reacted at 40°C for 20 min, the reaction solution was added dropwise to the LFD and incubated for different time periods. The results showed that the highest T/C ratio was obtained at 8 min of incubation (**Figures 3E, F**). The above results indicated that the biotin-labeled reporter reaction system was optimal for 20 min at 40°C and 8 min incubation with drops of LFD.

### 3.4 Specificity of the *KP177R* RPA-CRISPR/*Cas12a* assay

**Figure 4:**
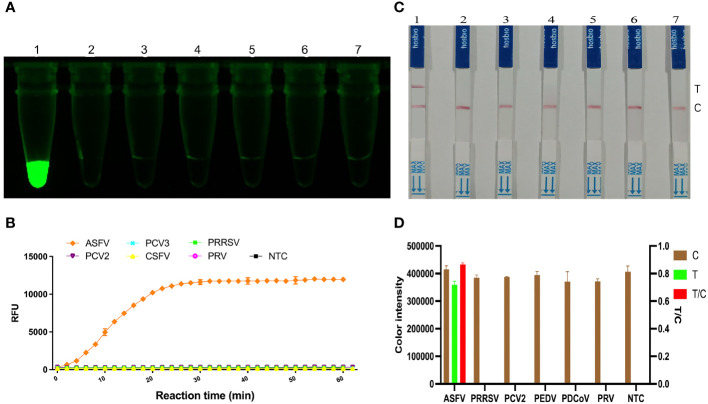
Specificity analysis of the *KP177R* RPA-CRISPR/*Cas12a* assay. **(A)** The visual fluorescein-labeled *KP177R* RPA-CRISPR/*Cas12a* assay was performed to detect six types of virus nucleotides, including ASFV, PRRSV, PCV2, PEDV, PDCoV, PRV, and the no-template control (NTC), marked with dipsticks 1−7, respectively. **(B)** fluorescein-labeled *KP177R* RPA-CRISPR/*Cas12a* assay was performed to detect the aforementioned six types of virus nucleotides in a microplate reader, showing their fluorescence intensities in relative fluorescence units (RFU). **(C)** The visual biotin-labeled LFD *KP177R* RPA-CRISPR/*Cas12a* assay was performed to detect the aforementioned six types of virus nucleotides, including ASFV, PRRSV, PCV2, PEDV, PDCoV, PRV, and the no-template control (NTC), marked with dipsticks 1−7, respectively **(D)** Image J densitometry of the signal intensities at the T- and C-lines in the LFD and calculation of their relative densitometric ratios for six types of virus nucleotides and NTC.

To determine the specificity of the *KP177R* RPA-CRISPR/*Cas12a* assay for ASFV, RPA amplification was performed for different viruses. Under blue light irradiation, only the ASFV nucleic acid template group emitted green fluorescence, while the rest of groups were non-fluorescent (**Figure 4A**); the microplate reader also showed fluorescent signals only in the ASFV template group when working at 37°C for 1 h in the assay (**Figure 4B**). Correspondingly, only the T-line of the ASFV group showed a blot in LDF, whereas the rest of the groups showed no blots (**Figures 4C, D**). These results indicate that the *KP177R* RPA-CRISPR/*Cas12a* assay has good specificity.

### 3.5 Sensitivity of the *KP177R* RPA-CRISPR/*Cas12a* assay

**Figure 5:**
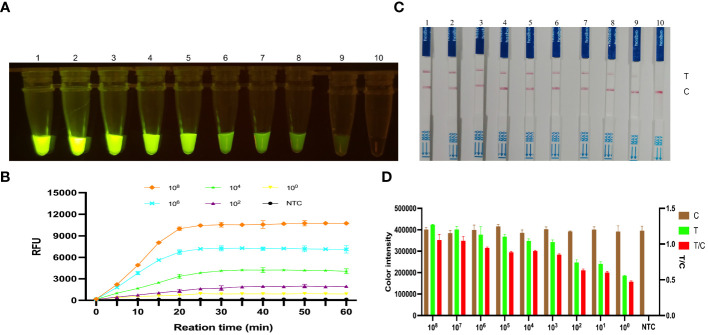
Sensitivity analysis of the *KP177R* RPA-CRISPR/*Cas12a* assay for ASFV detection. **(A)** The visual fluorescein-labeled *KP177R* RPA-CRISPR/*Cas12a* assay was performed to detect tenfold serial ASFV DNA dilutions. Dipsticks 1−10 represent 6.83 × 10^8^ – 6.83 × 10^0^ copies/μL ASFV DNA and the no-template control (NTC), respectively, and green fluorescence can be observed on the dipsticks 1−9 with the unaided eye. **(B)** The fluorescein-labeled *KP177R* RPA-CRISPR/*Cas12a* assay was performed to detect tenfold serial ASFV DNA dilutions in a microplate reader, with the relative fluorescence unit (RFU) to display the fluorescence intensity. The six lines represent 6.83 × 10^8^, 6.83 × 10^6^, 6.83 × 10^4^, 6.83 × 10^2^, 6.83 × 10^0^ copies/μL ASFV DNA and the NTC. **(C)** The visual biotin-labeled LFD *KP177R* RPA-CRISPR/*Cas12a* assay was performed to detect tenfold serial ASFV DNA dilutions. Dipsticks 1−10 represent 6.83 × 10^8^ – 6.83 × 10^0^ copies/μL ASFV DNA and the NTC, respectively. Similarly, red blots can be observed on the dipsticks 1−9. **(D)** Image J densitometry of the signal intensities at the T- and C-lines in the LFD and calculation of their relative densitometric ratios for serially diluted nucleic acid samples and NTC.

The *KP177R* positive plasmid pMD18-T-*KP177R* was tenfold diluted, which were used as templates for RPA amplification. Reaction systems were used to perform RPA-CRISPR/*Cas12a* assays on the different concentrations of DNA templates mentioned above. Under blue light irradiation, a visual green fluorescent response was observed in the tube with a minimum template concentration of 6.83 × 10^0^ copies per microliter (**Figure 5A**). Similarly, the fluorescence signal was detected in the tube with a minimum concentration of 6.83 × 10^0^ copies per microliter in a microplate reader (**Figure 5B**). Correspondingly, the appearance of T-line red blot and T/C ratios were observed at a minimum of 6.83 × 10^0^ copies per microliter of template for the LFD (**Figures 5C, D**). These results indicate that the *KP177R* RPA-CRISPR/*Cas12a* fluorescein- and biotin-labeled reaction system is highly sensitive, with a detection limit of 6.83 × 10^0^ copies per microliter of viral nucleic acid.

### 3.6 Reproducibility of the *KP177R* RPA-CRISPR/*Cas12a* assay

**Figure 6:**
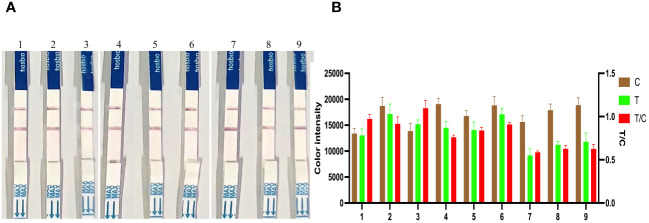
Reproducibility analysis of the *KP177R* RPA-CRISPR/*Cas12a* assay. **(A)** Dipsticks 1−3, RPA amplification product of 6.83 × 10^7^ copies/μL of strongly positive *KP177R* DNA was tested at the first, second, and third months, respectively, and the signal intensity of the T-line did not change over the three months. Similarly, dipsticks 4−6 and dipsticks 7−9 represent 6.83 × 10^5^ and 6.83 × 10^3^ copies/μL of moderately and weakly positive *KP177R* gene DNA, respectively, and the signal intensity of the T-line did not change over the three months. **(B)** Image J densitometry of the signal intensities at the T- and C-lines in the LFD and calculation of their relative densitometric ratios.

RPA amplification products of 6.83 × 10^7^, 6.83 × 10^5^, and 6.83 × 10^3^ copies/μL of strongly, moderately, and weakly positive *KP177R* gene were used to determine the reproducibility of the CRISPR/*Cas12a* assay. The signal intensities at the T line generated by the same ASFV DNA concentration did not obviously change over the three months of sampling (**Figure 6**). These results showed that the *KP177R* RPA-CRISPR/*Cas12a* assay had good reproducibility.

### 3.7 Validation of the performance of the *KP177R* RPA-CRISPR/*Cas12a* assay for ASFV detection using clinical samples

**Figure 7:**
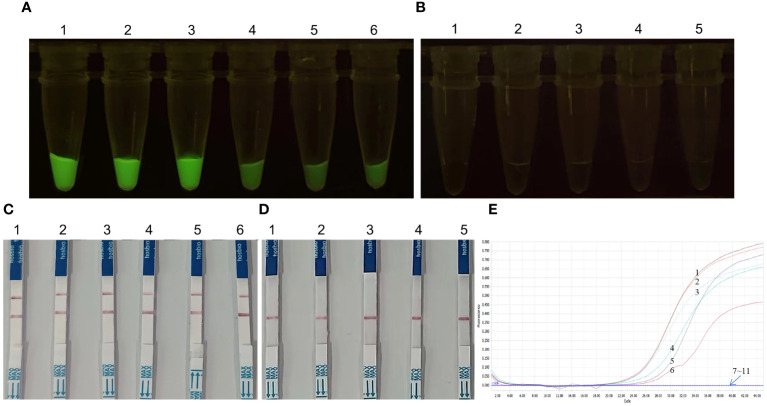
*KP177R* gene fragments amplified from clinical samples were detected by the *KP177R* RPA-CRISPR/*Cas12a* assay and RT-qPCR for ASFV. **(A)** Ten *KP177R* gene fragments were detected by fluorescein-labeled *KP177R* RPA-CRISPR/*Cas12a*, tubes 1−6: show positive green fluorescence. **(B)** tubes 1−4 show no fluorescent negatives and tube 5 is the no-template control (NTC). **(C)** Ten *KP177R* gene fragments were detected by biotin-labeled *KP177R* RPA-CRISPR/*Cas12a*, dipsticks 1−6: show positive red blot. **(D)** dipsticks 1−4 show no red blot negatives and dipstick 5 is the NTC. **(E)** Ten clinical samples were detected by RT-qPCR, curves 1−6 represent the positive results, 7−10 represent the negative results, and 11 is the NTC.

Ten *KP177R* gene fragments were amplified from clinical samples and detected using fluorescein- and biotin-labeled *KP177R* RPA-CRISPR/*Cas12a* assays and RT-qPCR. The results of fluorescein-labeled *KP177R* RPA-CRISPR/*Cas12a* showed that 6 samples tested positive, while 4 samples tested negative (**Figures 7A, B**), and the above-mentioned six positive and four negative gene fragments in the biotin-labeled *KP177R* RPA-CRISPR/*Cas12a* assay also showed identical positive and negative results, respectively (**Figures 7C, D**). Fluorescein- and biotin-labeled *KP177R* RPA-CRISPR/*Cas12a* were used to test each *KP177R* gene fragment, and the results were compatible. Similarly, these ten samples were detected by RT-qPCR, the *KP177R* gene fragments could not be detected by qPCR recommended by WOAH, which target on B646L gene (p72). The diagnostic coincidence rate for both assays was 100% and the kappa value was 1.000 (*p* < 0.001). The viral nucleic acid contents of these six positive samples were 10^3.97^, 10^3.81^, 10^3.36^, 10^2.93^, 10^2.59^, and 10^2.49^ copies per microliter, respectively (**Figure 7E**).

## 4 Discussion

ASF was first reported in Kenya in 1921, and over the years has ravaged most countries in Africa, Europe, and the Americas. It was later introduced to Russia via Central Asia and the Caucasus, causing severe economic losses to the global pig industry (16, 17). This disease previously has been reported in 37 countries. Entering 2023, the ASF outbreak is still occurring in many parts of the world. In April, several countries experienced ASF including Hungary, Poland, Italy, Germany, Russia, Romania, Nepal, and South Korea. In August, some countries such as Serbia, Latvia, Croatia, Hungary, Moldova, Bosnia and Herzegovina, Russia, Ukraine, Italy, North Macedonia, Germany, and Poland also reported ASF cases. In November, a number of countries such as Italy, Poland, Russia, Germany, Croatia, Ukraine, Sweden, Romania, Latvia, and Hungary continued to be affected by ASF (18). As a major global pig producer and trading country, China is in the context of a gradually increasing pig industry and has a close trade flow of pigs with the international community. The task of prevention and control of ASF is still very difficult because of the diverse transmission routes of ASFV, the continuous emergence of different genotypic strains and new mutant strains, which leads to an increasingly complex epidemiological situation of the disease, and the fact that there is still no credible commercial vaccine (19–21).

To detect viruses in the environment and infected pigs in the first instance and thus achieve the goal of ASF prevention, control, and elimination, it is crucial to develop detection assays that are suitable for little instrumentation and equipment and do not require specialized technicians. To reduce the dependence on thermal cycling instruments and skilled operators and to amplify gene fragments at lower temperatures with simple heating equipment, the present study adopted the isothermal amplification of nucleic acids, which has emerged in recent years. Representative isothermal amplification techniques include loop-mediated isothermal amplification (LAMP) and recombinase-mediated isothermal amplification (RAA/RPA). LAMP has been developed and applied earlier, but it requires higher reaction temperatures (more than 60°C), and multiple primers, which affects the popularization of the technique (22). Compared with the LAMP assay, the RPA technology developed in recent years can complete the whole experimental process at about 37°C, which is not a high-temperature requirement and can be easily achieved in farms and other occasions. The temperature of the reaction is close to the human body temperature. In certain underdeveloped regions, in the absence of experimental equipment, nucleic acid amplification can still be carried out effectively at human body temperature as demonstrated previously (23). Hence, the RPA technology is more suitable for the needs of the low requirements of the heating equipment.

In this study, we exploited the high specificity of the CRISPR/Cas system by cutting target genes to improve its sensitivity for the determination of viral nucleic acids. In recent years, the *Cas12a* protein, which has been widely used, has a simple structure and a single promoter that can initiate multiple crRNAs simultaneously, making the CRISPR/*Cas12a* editing system more advantageous in terms of structure and mechanism of action, and it has been widely used in the detection of nucleic acids of pathogens (24). For example, some researchers have used the CRISPR/*Cas12a* system combined with RPA isothermal amplification technology to detect human papillomavirus (HPV) and encephalitis B virus, which has simplified the detection process and shortened the detection time, while greatly improving detection accuracy (25, 26). Several studies have reported the molecular detection techniques for ASFV, including RPA (27–29), RPA combined with RT qPCR (30), RPA combined with CRISPR/Cas (31), and LAMP combined with CRISPR/Cas12a (32). Some of them only use a single RPA or LAMP amplification technique to establish detection, while others establish joint methods with RT-qPCR or CRISPR/Cas, but these joint methods also only use fluorescence staining or colorimetric analysis without using test strip staining. The detection limit of viral DNA for these assays’ ranges from as low as three point five copies per microliter (27) to as high as five hundred and eighty copies per microliter (32).

Lb*Cas12a* from the Trichosporonaceae family was used as a template (33, 34), whereas the *Cas12a* protein was expressed by a prokaryotic expression system and its activity was determined after purification. ssDNA reporter molecules broke in the presence of this protein, demonstrating that this protein has good cleavage efficiency and can be used to set up a reaction system for the detection of ASFV nucleic acids (35, 36). The CRISPR/*Cas12a* reaction system established in this study showed different optimal reaction temperatures in the fluorescein-labeled reporter reaction system, which was 37°C, and the biotin-labeled reporter reaction system, which was 40°C, suggesting that the cleavage efficiency of the *Cas12a* protein could be affected by the different marker molecules. However, after the reaction conditions were optimized, the two reaction systems showed the same detection sensitivity with different reaction temperatures. The detection limit for both the fluorescein- and biotin-labeled CRISPR/*Cas12a* reaction systems in this study was 6.8 copies per microliter, which is approach to the detection limits (sensitivities) of 6 copies per microliter as reported previously (27, 32). This result suggests that the detection sensitivity of RPA-assisted RT-qPCR was higher than that of LAMP-assisted CRISPR/*Cas12a*. The former requires RT-qPCR or colorimetric analysis after the RPA reaction, which requires complex instrumentation and specialized technicians, whereas the latter facilitates the operation but has low sensitivity and reduces the detection rate of pathogens (37). The RPA-assisted CRISPR/*Cas12a* assay established in this study has the advantage of convenience and achieves high detection sensitivity. At the same time, we also recognize the limitation of this study, that is, the sample was 10-fold diluted, and if they were further diluted 2-fold to 3.4, even 1.7, the LOD may be lower than 6.83 in the *KP177R* RPA-assisted CRISPR/*Cas12a* assay.

In the CRISPR/Cas system, Cas proteins are guided by gRNAs, and the recognition of protospacer adjacent motifs (PAM) sequences enables the binding of Cas proteins to the target dsDNA, activating their cleavage activity (38, 39). The designed gRNA specifically binds to the target sequence; therefore, sequence selection is crucial. In this study, we designed the gRNA based on the protospacer sequence following PAM. The highly conserved *KP177R* target region of ASFV was used as a template. The TTTN sequence was used as the starting point, 20 nt was selected backward, and a hairpin structural sequence was added in front of this fragment, leading to better specific recognition of the target sequence. The process of designing RPA primers must be performed according to the binding site of the gRNA, and the designed primers must contain the sequences of the targets to be detected (40). The length of the RPA primers should preferably be 30−35 bp; otherwise, recombinase activity will be affected. Consecutive polyguanine should be avoided at the 5’ end so as not to interfere with the recombination of the amplified fragments; cytosine and guanine are preferred at the 3’ end to improve the amplification performance of the primers (41, 42). At the same time, screening elimination was needed to determine the most suitable primers for RPA; the more primers screened, the greater the chance of finding the most suitable primers for RPA (43).

Particularly, the molecular detection methods described in previous reports are always focus on CP204L and B646L genes; while the KP117R gene was not applied as detection target. We assessed the 120 bp length fragment sequences of the *KP177R* gene from a total of 100 ASFV strains isolated from Africa (4), Europe (38), Central Asia, Russia (14), North America (1), East Asia (38), Southeast Asia (3), and South Asia (2). The nucleotide sequences of 120 bp length fragment from the 100 viral strains were identical to those of the ASFV China/2018/Anhui XCGQ strain with 100 (91 strains), 98.3 (one strain), 95 (seven strains), and 94.2% (one strain) homology (**Supplementary Figure 1**). Among them, there were 28 Chinese strain sequences, 23 of which had 100% homology with ASFV China/2018/AnhuiXCGQ and belonged to genotype II, and the other five belonged to genotype I strains or recombinant strains of genotypes I and II. Moreover, the *KP177R* gene has been shown that it does not affect the virus replication and virulence, and it is promising to be used in gene deletion vaccines. Thus, it can be concluded that the *KP177R* gene is highly conserved in nucleotide sequence and is suitable as a detection target for the future candidate ASFV vaccines.

In this study, we established an ASFV nucleoid acid detection assay targeting *KP177R* by integrating RPA amplification and CRISPR/*Cas12a* precision gene cutting. This assay is rapid and requires neither specialized equipment nor highly qualified personnel. The detection results can be determined by the visual pathway of fluorescence or LFD coloration. The *KP177R* RPA-CRISPR/*Cas12a* assay is especially suitable for swine farms or clinical sample testing sites with poor experimental conditions and can provide valuable technical support for the rapid screening of ASFV.

## Data availability statement

The original contributions presented in the study are included in the article/**Supplementary Materials**. Further inquiries can be directed to the corresponding authors.

## Author contributions

HL: Methodology, Writing – original draft. SW: Methodology, Writing – original draft. LJ: Writing–review & editing. TL: Formal Analysis, Methodology, Writing – original draft. HS: Validation, Writing – original draft. SG: Investigation, Writing – original draft. SJ: Validation, Writing – review & editing. JW: Visualization, Writing – review & editing. JP: Conceptualization, Funding acquisition, Project administration, Supervision, Writing – review & editing.
